# Pathological Eating Patterns in Adults Displaying Obsessive‐Compulsive Symptoms: A Scoping Review

**DOI:** 10.1002/erv.70071

**Published:** 2025-12-23

**Authors:** Sonay Kucukterzi‐Ali, Amanda K. Ludlow, Roberto Gutierrez, Naomi A. Fineberg, Tim M. Gale

**Affiliations:** ^1^ Department of Psychology, Sport and Geography University of Hertfordshire Hatfield UK; ^2^ University of Sussex Brighton UK; ^3^ Hertfordshire Partnership University Foundation NHS Trust Hatfield UK; ^4^ Department of Clinical, Pharmaceutical and Biological Science University of Hertfordshire Hatfield UK

**Keywords:** adults, eating behaviours, eating disorders, obsessive compulsive disorders

## Abstract

**Aim:**

Numerous studies have now shown that those with eating disorders have statistically higher rates of obsessive‐compulsive disorder (OCD). However, much less is known about the eating behaviours of adults with OCD. This scoping review aimed to identify and synthesise the existing literature describing pathological eating behaviours in adults with OCD and/or obsessive‐compulsive (OC) symptoms.

**Method:**

The databases PubMed, Scopus, ScienceDirect and PsychNet were searched in accordance with Preferred Reporting Items for Systematic reviews and Meta‐Analyses (PRISMA) guidelines.

**Results:**

Fifty‐nine studies met the eligibility criteria and were included in this review. There was a high occurrence of eating disorders identified in both adults with a diagnosis of OCD and in those with elevated OC symptoms. The literature appears inconclusive as to which eating disorders most commonly co‐occur alongside OCD. Furthermore, it was noted that a number of adults with OCD and those with elevated OC symptoms showed high levels of eating behaviours falling below clinical thresholds, including behaviours synonymous with avoidant‐restrictive food intake disorder and orthorexia nervosa.

**Conclusions:**

Future research needs to identify underlying factors for these problematic eating behaviours in OCD to adapt current treatment modalities to meet this group's needs. Addressing particular subsets of adults with OCD and/or OC symptoms, such as those with co‐occurring anxiety and mood disorders, may be key to understanding who are most at risk.

**Trail Registration:**

The scoping review was registered on OSF: https://osf.io/s8kan

## Highlights

- OC symptoms are associated with a broad range of eating difficulties, including dieting‐related disorders, binge‐eating, ARFID, and some non‐clinical eating patterns
- Different OC symptom types (e.g., checking, contamination, hoarding) show distinct links to specific eating patterns
- Transdiagnostic factors, such as perfectionism and emotion dysregulation, may explain atypical eating in OCD, though the exact nature of this relationship remains unclear.

## 1 Introduction

Obsessive‐Compulsive Disorder (OCD) is a psychiatric condition characterised by intrusive, anxiety‐evoking thoughts and repetitive acts (American Psychiatric Association 2013). It affects around 1%–3% worldwide (Angst et al. 2005), with the prevalence of obsessive‐compulsive symptoms (OC) in the general population considered to be around five times higher (Ruscio et al. 2010). The exact prevalence of OC symptoms is difficult to determine as they frequently co‐occur with many other psychiatric conditions that present with overlapping features, including body dysmorphic disorder, Tourette syndrome, autism spectrum disorder, and eating disorders (e.g., anorexia nervosa; Brock et al. 2025). In particular, eating disorders and OCD share several important characteristics, including the heightened need for control and the presence of repetitive, intrusive thoughts, with co‐occurrence estimated to be as high as 41% (Kaye et al. 2004; Sallet et al. 2010; B. M. Williams and Levinson 2022).

Research has demonstrated that those with eating disorders, including anorexia nervosa, bulimia nervosa and binge‐eating disorder, may additionally present with a co‐occurring OCD diagnosis or elevated levels of OC symptoms (e.g., Breithaupt et al. 2014; Kambanis et al. 2020; van Passel et al. 2020). Similar patterns have also been observed among those with OCD, whereby those affected are more likely to demonstrate non‐clinical eating behaviours or experience a co‐occurring eating disorder (e.g., Assunção et al. 2012; Bang et al. 2020; Garcia et al. 2020). Whilst an association between OCD and pathological eating patterns has been observed, most of the research to date has centred on the presence of OCD and OC symptoms in those presenting with an eating disorder, particularly those associated with dissatisfaction with weight and body shape (e.g., anorexia nervosa). Consequently, less is known about whether other eating behaviours manifest in OCD or those with OC symptoms; for example, avoidant‐restrictive food intake disorder (ARFID), in which avoidance is characterised by a lack of interest or appetite for food, fear of adverse consequences associated with eating, and/or aversive sensory experiences related to food.

Limitations in our understanding of the relationship between OC symptoms and eating behaviours also extend to non‐clinical eating behaviours, which vary in severity and are less likely to require clinical intervention. For example, non‐clinical eating behaviours, such as selective eating and restrictive eating symptoms, have both been linked to increased levels of OC symptoms (e.g., Barnhart et al. 2021; Pollack and Forbush 2013; Wildes et al. 2012). While food selectivity, coined for the inadequate variety and consumption of food, is not currently recognised as a clinical concern (Kerzner et al. 2015), if left untreated, it can lead to adverse feeding concerns, including nutritional deficiencies (Fildes et al. 2015; Galloway et al. 2003; Galloway et al. 2005), and an increased risk of developing an eating disorder or OC symptoms (Herle et al. 2020; Zickgraf et al. 2020; Zohar et al. 2025). Moreover, eating behaviours leading to food avoidance or restriction are also associated with weight loss and slower growth development (Tanner and Richmond 2024; Taylor and Emmett 2018). Severe levels of food selectivity in adulthood are associated with less enjoyment of eating (Kauer et al. 2015) and greater impairment in quality of life related to eating (Wildes et al. 2012). Hence, it would be important to address such eating behaviours among those with OCD or those showing high levels of OC symptoms.

This scoping review aimed to provide an overview of the relationship between OC symptoms and pathological eating behaviours, including eating disorders and non‐clinical eating behaviours, in both the general population and OCD population, and to identify gaps in the current literature base.

## 2 Method

### 2.1 Review Methodology

A scoping review was proposed to address the types of pathological eating behaviours found in adults with OCD and in individuals from the general population displaying high levels of OC symptoms. This review methodology was chosen as, although studies examining atypical eating in individuals with OC symptoms do exist, the evidence base is limited, largely surface‐level in depth, and characterised by heterogeneous methodological approaches. The objective of the review was to map what is currently known about atypical eating in this population and to identify gaps that may guide future empirical research, rather than to conduct a comparative synthesis.

Guidelines from the JBI methodology for scoping reviews (Aromataris et al. 2024) and Preferred Reporting Items for Systematic Reviews and Meta‐Analyses guidelines (PRISMA; Tricco et al. 2018) were used to conduct the proposed scoping review. The scoping review has been registered on OSF: https://osf.io/s8kan. No ethical approval was required for this review and no funding was received to conduct the review.

### 2.2 Eligibility Criteria

The articles considered for inclusion in the review were required to examine eating behaviours, including eating disorder symptoms or non‐clinical eating behaviours, and consider their relationship with OC symptoms or presence in OCD. Participants needed to be from the clinical OCD population or the general population and have (i) a diagnosis of OCD and/or (ii) have completed a measure of OCD and/or OC symptoms. Studies could adopt qualitative and/or quantitative methodologies. There were no restrictions on the geographical location of the research. Given the exploratory nature of the review, research articles were only excluded if they were systematic reviews, book chapters, case studies, did not include human participants, or were not published in English. Studies not relevant to the literature review aim were also excluded. Furthermore, studies had to be published from 2013 onwards, which marked the introduction of the *Diagnostic and Statistical Manual of Mental Disorders* (fifth ed.; APA, 2013).

### 2.3 Literature Search Strategy

The literature search was conducted using PubMed, Science Direct, Scopus and PsychNet databases with the following search terms:

(‘obsessive‐compulsive disorder’ OR ‘OCD’ OR ‘obsessive‐compulsive symptoms’) AND (‘problematic eating’ OR ‘abnormal eating’ OR ‘binge‐eating’ OR ‘disinhibited eating’ OR ‘disturbed eating’ OR ‘external eating’ OR ‘dysregulated eating’ OR ‘emotional eating’ OR ‘maladaptive eating’ OR ‘restrictive eating’ OR ‘orthorexia nervosa’ OR ‘anorexia nervosa’ OR ‘bulimia nervosa’ OR ‘binge‐eating disorder’ OR ‘picky eating’ OR ‘selective eating’ OR ‘food neophobia’ OR ‘avoidant‐restrictive food intake disorder’)

Search terms were kept broad to capture the breadth of eating behaviours which may present in OCD or alongside elevated OC symptoms. In line with guidance from Haddaway et al. (2015), the first 200 results from Google Scholar were also screened for eligibility. Moreover, reference lists of eligible articles were manually scanned for relevant articles. Grey literature sources (e.g., dissertations, theses, preprints) were not included in the search.

The literature search was initially carried out in 2022 and then updated in March 2025. Literature databases were continually monitored for new articles. Following searches on each literature database, all citations were exported to Rayyan, an online literature review tool, to remove duplicates and identify eligible articles (Ouzzani et al. 2016). An initial screen of article titles and abstracts was carried out, after which full‐text articles were assessed for eligibility.

### 2.4 Study Selection

Searches resulted in 8270 records. Prior to screening, 2024 duplicate records were removed, leaving a total of 6246 articles to be screened. Abstract screening was conducted by the first author. Ambiguous cases were discussed with a second author until an agreement was reached; if consensus could not be reached, a third author reviewed the article to determine eligibility. After the titles and abstracts were screened, 201 articles remained for the full‐text screening. Of these articles, 96 were eligible for the scoping review.

**FIGURE 1:**
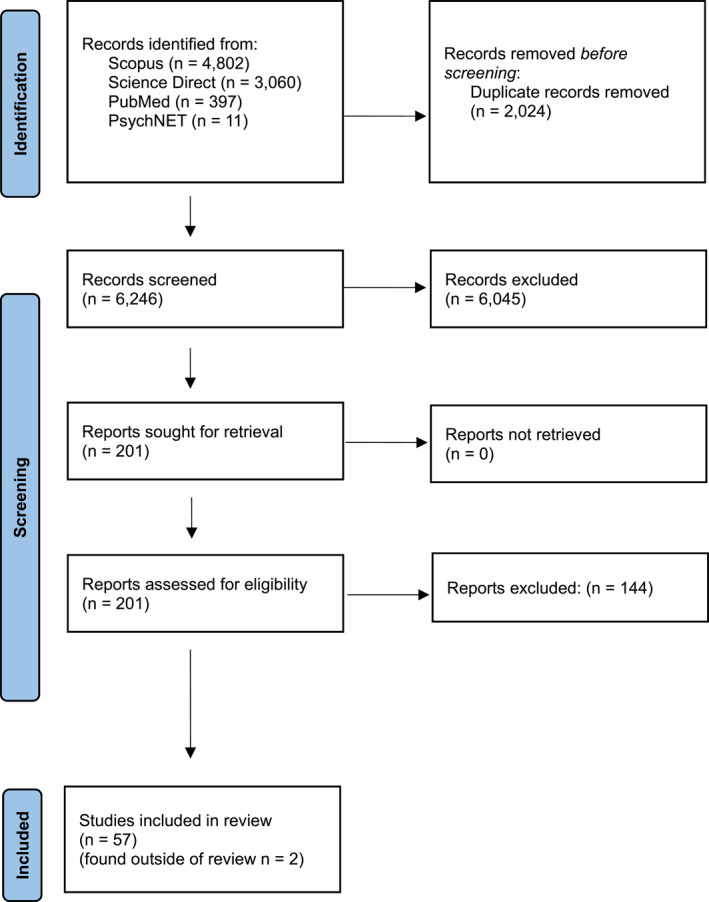
PRISMA diagram.

Due to the volume of articles eligible, the inclusion criteria were further refined to include only studies of adults. In total, eight studies of children and adolescents were excluded from eligible articles. Moreover, studies with research questions beyond the scope of pathological eating patterns and OC symptoms, or psychopathologies including OC symptoms (e.g., anxiety and depression, alongside OC symptoms), were excluded. For example, if a study adopted measures of pathological eating behaviours and/or OC symptoms, but did not aim to examine their relationship, it was not included in the review. After refining the inclusion criteria, a total of 59 studies were deemed eligible for the review; 57 of these studies were found during the literature search, and two studies were found outside of the search. The study selection process is reported in Figure 1 using the PRISMA extension for Scoping Reviews (Tricco et al. 2018) and a summary of eligible studies is provided in Tables 1 and 2.

**TABLE 1:** Summary of OCD population studies.

Author(s) (year of publication)	Participants, gender, age, diagnosis and country of research	Purpose	OC symptom and eating measures used	Findings
Torresan et al. (2013)	OCD *N =* 858 Females *n* = 504 (58.7%) Age *M =* 35.4, SD *=* 12.1 Brazil	Examine comorbidity in OCD	Yale‐Brown Obsessive Compulsive Scale Structured Clinical Interview for Diagnosis of Axis I DSM‐IV Disorders	12.8% prevalence rates of eating disorders in OCD In general, females with OCD were more likely to have an eating disorder compared to males with OCD, particularly anorexia nervosa and bulimia nervosa. There was no difference in rates of binge‐eating disorder between males and females
Cederlöf et al. (2015)	OCD *n =* 19,814 Females *n* ≈ 11,195 (56.5%) Anorexia nervosa *n =* 8462 Females *n* ≈ 7920 (93.6%) Adult participants, ages not provided Sweden	Longitudinal study to explore the overlap between OCD and anorexia nervosa	National Patient Register (Sweden)	Those with antecedent OCD had a four‐fold risk of developing anorexia nervosa at follow up; this risk was greater for males (16‐fold risk for females, 37‐fold risk for males) Those with prior anorexia nervosa had a 10‐fold risk of developing OCD
Poyraz et al. (2015)	OCD *n =* 49 Females *n* = 36 (73.5%) Age *M =* 31.37, SD *=* 10.97 Panic disorder *n =* 44 Females *n* = 30 (68.2%) Age *M =* 33.43, SD *=* 9.96 Generalised anxiety disorder *n =* 37 Females *n* = 31 (83.8%) Age *M =* 35.03, SD *=* 9.58 Türkiye	To investigate the relationship between orthorexia nervosa and OC symptoms in OCD, generalised anxiety disorder and panic disorder	Padua Inventory Washington State University Revision Eating Attitudes Test—40 ORTO‐11	Eating disorder symptoms and orthorexia nervosa did not differ between those with OCD, panic disorder and generalised anxiety disorder OC symptoms were associated with orthorexia nervosa symptoms
Tyagi et al. (2015)	OCD *n =* 135 Non‐OCD anxiety disorders *n =* 44 Age of entire sample *M =* 37.5, SD *=* 13.5 Gender of entire sample Females *n* ≈ 98 (54.8%) England, UK	To compare the prevalence of eating disorders in OCD and other anxiety disorders	SCOFF Questionnaire International Classification of Diseases and Related Health Problems (10th Revision)	No significant difference in the prevalence rate of eating disorders between OCD and other anxiety groups
M. T. Williams et al. (2017)	OCD *n =* 75 Females *n* ≈ 42 (56.8%) Age *M =* 41.4, SD *=* 12.3 USA	To explore comorbidities of OCD in underrepresented populations	Structured Clinical Interview for Diagnosis of Axis I DSM‐IV Disorders Yale‐Brown Obsessive Compulsive Scale	4.1% of OCD participants had binge‐eating disorder at some point during their lifetime Anorexia nervosa and bulimia nervosa were not observed among participants
Ay and Aytas (2018)	OCD *n =* 60 Gender split* *n* = 31/29 Age *M =* 31.33, SD *=* 9.44 Healthy controls *n =* 60 Gender split* *n* = 31/29 Age *M =* 31.10, SD *=* 6.92 *Gender not reported Türkiye	To investigate the eating disorder attitudes in OCD and explore the relationship between distress tolerance and eating attitudes	The Padua Inventory Eating Attitudes Test—26 Distress Tolerance Scale	OCD participants had significantly higher eating disorder symptoms and poorer distress tolerance compared to controls OCD participants with greater eating disorder symptoms had more severe overall OC symptoms OC symptoms correlated with eating disorder symptoms; eating disorder symptoms associated with poorer distress tolerance
Hofer et al. (2018)	General population *n =* 3021 of which, *n* = 55 met OCD criteria Participants followed up between ages of 14–24 years Details of gender not provided Germany	To examine whether OCD is a risk for mental health disorders	Munich‐Composite International‐Diagnostic Interview	OCD was associated with the development of bulimia nervosa, but not anorexia nervosa In those diagnosed with OCD, attributable fractions demonstrated 85.3% of bulimia nervosa cases were linked to the OCD
Peters et al. (2019)	Anxiety disorders *n =* 7221, of which OCD *n =* 83 Females (OCD) *n* ≈ 50 (60.5%) Age (OCD) *M =* 37.6, SD *=* 2.4 Canada (data obtained from England, UK)	To examine the effect of mood instability on the relationship between anxiety disorders and self‐injury and binge‐purge behaviours	Clinical Interview Schedule‐Revised Structured Clinical Interview for DSM‐IV Axis II Personality Disorders	12.8% of OCD participants engaged in binge‐purge behaviours OCD could predict binge‐purge behaviours, but this effect reduced when considering mood instability and impulsivity, particularly mood instability
Bang et al. (2020)	OCD *n =* 132 Females *n* = 94 (71.2%) Age *M =* 33.92, SD *=* 8.95 Healthy controls *n =* 260 Females *n* = 233 (89.6%) Age *M =* 32.44, SD *=* 5.75 Norway	To examine the prevalence of eating disorders in OCD participants and controls	Obsessive‐Compulsive Inventory—Revised Eating Disorder Examination Questionnaire	Eating disorder symptoms did not differ between OCD participants and controls Female OCD participants were more likely to meet the threshold for an eating disorder compared to healthy controls
Yılmaz et al. (2020)	OCD *n =* 63 Females *n* = 40 (63.5%) Age *M =* 34.70, SD *=* 10.50 Healthy controls (non‐exercisers) *n =* 63 Females *n* = 35 (55.6%) Age *M =* 32.75, SD *=* 12.58 Healthy controls (exercisers) *n =* 63 Males *n* = 35 (55.6%) Age *M =* 29.00, SD *=* 7.85 Türkiye	To explore orthorexia nervosa symptoms in those with OCD, as well as healthy controls who engage in exercise	Yale‐Brown Obsessive Compulsive Scale Eating Attitudes Test—40 ORTO‐11	Eating disorder symptoms in OCD and the healthy control exercise group did not differ, but were greater than the healthy control non‐exercise group The healthy control exercise group had greater orthorexia nervosa symptoms compared to the OCD group; OCD group and the healthy control non‐exercise group did not differ on orthorexia nervosa symptoms No correlation between OC symptoms and orthorexia nervosa in the OCD group In the OCD group, eating disorder symptoms were linked to orthorexia nervosa
Hessler‐Kaufmann et al. (2021)	OCD *n =* 152 Females *n* = 90 (59%) Age *M =* 33.1, SD *=* 15.9 Germany	To examine the prevalence of orthorexia nervosa symptoms among inpatients	Düsseldorf Orthorexia Scale	At admission, 2% of OCD patients had orthorexia nervosa at admission and 3.7% at discharge. This change was not significant
Kaczkurkin et al. (2021)	Anxiety or anxiety related disorders *N* = 329, of which OCD *n* = 131 Age (OCD) *M =* 29.77, SD *=* 11.30 Females (OCD) *n* ≈ 69 (52.9%) USA	To explore whether perfectionism and depression explains the relationship between OC symptoms and eating pathology	Obsessive‐Compulsive Inventory—Revised Clinical Impairment Questionnaire	Weak, positive correlation between OC symptoms and eating pathology OC symptoms could predict eating pathology Perfectionism and depression partially mediated the relationship between OC symptoms and eating pathology OC symptoms had a direct effect on eating pathology when controlling for perfectionism and depression, but the effect of OCI symptoms was reduced when including these factors Note: All analyses conducted on total sample, including OCD, anxiety spectrum disorders and major depressive disorder
Vaccari et al. (2021)	OCD *n =* 50 Females *n* = 16 (32%) Age *M =* 38.3, SD *=* 12.7 Anxiety spectrum disorders *n =* 42 Females *n* = 35 (83.3%) Age *M =* 46.2, SD *=* 13.7 Healthy controls *n =* 253 Females *n* = 135 (57.2%) Age *M =* 34.5, SD *=* 13.5 Italy	Explore the prevalence of orthorexia nervosa in OCD compared to participants with anxiety disorders and healthy controls	Obsessive‐Compulsive Inventory—Revised ORTO‐15	Prevalence of orthorexia nervosa was similar between OCD, anxiety disorders and healthy controls The OCD group did not have a greater risk for orthorexia nervosa compared to controls and anxiety disorders Those restricting their diet were more likely to display orthorexia nervosa symptoms
Danner et al. (2022)	OCD *n =* 419 Females *n* ≈ 233 (55.8%) Age *M =* 36.6, SD *=* 10.92 Netherlands	Investigate the relationship between OC symptoms and eating disorders in OCD	Padua Inventory‐Revised Yale‐Brown Obsessive Compulsive Scale Structured Clinical Interview for Diagnosis of Axis I DSM‐IV Disorders	10.5% of the OCD sample had a lifetime eating disorder Those with OCD and an eating disorder had more impulsive urges and depression and anxiety symptoms, compared to those without an eating disorder
Rai et al. (2022)	OCD *n =* 150 Females *n* = 52 (34.7%) Age *M =* 30.57, SD *=* 10.26 Healthy controls *n =* 131 Females *n* = 45 (34.4%) Age *M =* 31.92, SD *=* 10.15 India	To explore behavioural addictions, including food addictions, in OCD and healthy controls	Yale‐Brown Obsessive Compulsive Scale Eating Attitudes Test—26 (Bulimia and Food Preoccupation Subscale)	OCD participants and healthy controls had similar prevalence rates of food addiction (7.3% vs. 8.4%), but OCD participants reported more severe food addiction symptoms than healthy controls overall
Cosh et al. (2023)	Self‐identified dieters and/or having mental health symptoms *n =* 196 of which, *n* = 132 met OCD criteria Females (entire sample) *n* = 172 (87.3%) Non‐binary/withheld *n* = 8 (4.1%) Age (entire sample) *M =* 34.7, SD *=* 14.2 Australia	To examine the relationship between orthorexia nervosa and eating disorders and OCD	Obsessive‐Compulsive Inventory—Revised Eating Disorder Examination Questionnaire The Orthorexia Nervosa Inventory Eating Habits Questionnaire	Orthorexia nervosa symptoms could predict OC symptoms, but orthorexia nervosa had a stronger association with eating disorders rather than OCD Note: Findings are based on the total sample, in which 67% had OCD and 9.2% had probable anorexia nervosa. The remaining participants identified as dieting and/or having mental health symptoms but did not meet criteria for OCD or anorexia nervosa

*Note:* ≈ Gender *n* based on percentages provided by the authors.

**TABLE 2:** Summary of general population studies.

Author(s) (year of publication)	Participants, gender, age, diagnosis and country of research	Purpose	OC symptom and eating measures used	Findings
Pollack and Forbush (2013)	General population *n* = 407 Females *n =* 191 (46.9%) *M =* 38.24, SD *=* 13.51 USA	To examine why OC symptoms and eating disorder symptoms co‐occur	The Schedule of Compulsions, Obsessions and Pathological Impulses Eating Disorder Examination Questionnaire Eating Disorder Inventory‐3 The Three Factor Eating Questionnaire	OC checking, cleaning and rituals predicted body dissatisfaction Checking, cleaning and pathological impulses (negatively) predicted restraint Checking predicted binge‐eating Perfectionism and neuroticism mediated the relationship between certain OC symptoms and eating disorder symptoms
Asil and Sürücüoğlu (2015)	Dieticians *n* = 117 Females *n* = 101 (86.3%) Age *M =* 34, SD *=* 11.2 Türkiye	To examine orthorexia nervosa in dieticians	Maudsley Obsessive Compulsive Inventory Eating Attitudes Test—40 ORTO‐15	OC symptoms were not correlated with orthorexia nervosa, but those with orthorexia nervosa had higher OC symptoms compared to those without
Kauer et al. (2015)	Picky eaters *n =* 16 Age *M =* 22.56, SD *=* 5.92 Females *n* ≈ 9 (56%) Non‐picky eaters *n =* 18 Age *M =* 31.00, SD *=* 9.77 Females *n* ≈ 12 (65%) USA	Examine correlates of picky eating in adults	The Padua Inventory	Picky eaters had greater OC symptoms than non‐picky eaters Picky eater status could predict OC symptoms
Belloch et al. (2016)	OCD risk group *n =* 92 Females *n* ≈ 64 (69.6%) Age *M =* 23.12, SD *=* 4.83 Eating disorder risk group *n =* 41 Females *n* ≈ 33 (80.5%) Age *M =* 23.22, SD *=* 4.90 No‐risk group *n =* 100 Females *n =* 69 (69.0%) Age *M =* 23.50, SD *=* 4.58 Spain	To explore OC and eating disorder related intrusive thoughts between those at risk for OCD or an eating disorder	The Clark‐Beck Obsessive Compulsive Inventory Eating Attitudes Test—26 Obsessional Intrusive Thoughts Inventory Eating Intrusive Thoughts Inventory	Those at risk of OCD had more intrusive thoughts about eating compared to controls, but less than those at risk of an eating disorder
Bundros et al. (2016)	Students *n* = 448 Females *n* = 325 (72.5%) Age *M =* 22.17, SD *=* 4.83 USA	To explore the relationship between orthorexia nervosa assessed by the Bratman Orthorexia Test and validated measures of eating, body dysmorphia and OC symptoms	Obsessive‐Compulsive Inventory—Revised Eating Attitudes Test—26 Bratman Orthorexia Test	OC symptoms were associated with eating disorder symptoms and orthorexia nervosa symptoms
Schulte (2016)	Students *n* = 236 Females *n =* 152 (64.6%) *M =* 19.78, SD *=* 1.45 United Arab Emirates	To examine predictors of binge‐eating in students from the United Arab Emirates	Obsessive‐Compulsive Inventory—Revised The Binge Eating Scale The Emotional Eating Scale The Weight‐ and Body‐Related Shame and Guilt Scale	Greater OC symptoms correlated with binge‐eating, but OC symptoms could not predict binge‐eating when controlling for other factors, such as emotional eating
Zickgraf et al. (2016)	General population *n* = 325 Females *n* ≈ 164 (50.5%) *M =* 33.92, SD *=* 10.54 Picky eating support group *n* = 81 Females *n* ≈ 57 (70.3%) *M =* 40.42, SD *=* 13.31 USA	To compare picky eaters with and without ARFID symptoms to those with disordered eaters and typical eaters	Obsessive‐Compulsive Inventory—Revised Eating Attitudes Test—26 Single question to assess picky eating—are you a picky eater? Author developed ARFID questionnaire	Typical eaters had the lowest OC symptoms Disordered eating participants and ARFID participants had similarly high levels of OC symptoms ARFID participants expressed similar levels of OC symptoms to selective eaters
Brytek‐Matera et al. (2017)	Students *n* = 120 Females *n* = 83 (69.2%) Age *M =* 22.74, SD *=* 7.31 Italy	To examine orthorexia nervosa symptoms and psychopathology in students	Maudsley Obsessive‐Compulsive Questionnaire ORTO‐15 Eating Attitudes Test—26	Greater OC symptoms were associated with fewer orthorexia nervosa symptoms in females No relationship between OC symptoms and orthorexia nervosa was observed in males
Hayes et al. (2017)	Students *n* = 404 Females *n* ≈ 334 (82.7%) *M =* 20.71, SD *=* 4.36 USA	Examine correlates of orthorexia nervosa	Obsessive‐Compulsive Inventory—Revised ORTO‐15 Bratman Orthorexia Self‐Test	A weak association was found between OC symptoms associated with greater orthorexia nervosa symptoms (ORTO‐15) Those with elevated orthorexia symptoms had greater OC symptoms compared to those with not elevated orthorexia nervosa
Fox et al. (2018)	Picky eaters *n* = 13 Females *n* = 11 (84.6%) Age *M =* 36.75 England, UK	Qualitative study to explore the lived experience of picky/selective eating	Semi‐structured interview	Preferred foods described as safe; unsafe foods described as a source of anxiety
Gezer and Yalvaç (2018)	University students *n =* 1754 Females *n* = 760 (43.3%) Age *M =* 21.3, SD *=* 2.3 Males *n* = 994 (56.7%) Age *M =* 22.2, SD *=* 2.6 Turkish Republic of Northern Cyprus	Examine eating behaviours and OC symptoms in students	Eating Attitudes Test—40 Maudsley Obsessive‐Compulsive Inventory	Positive, weak correlations observed between OC symptoms and eating disorder symptoms Those at risk of an eating disorder had significantly greater OC symptoms compared to those without such risk
Kim et al. (2018)	Females from general population *n =* 463, of which were identified as: Overweight females with binge‐eating disorder *n =* 22 Age *M =* 22.68, SD *=* 2.96 Normal weight females with binge‐eating disorder *n =* 29 Age *M =* 21.83, SD *=* 1.94 Korea	To explore determinants of binge‐eating disorder in normal and overweight women	Obsessive‐Compulsive Inventory—Revised Eating Disorder Examination Questionnaire Dutch Eating Behaviour Questionnaire	Normal weight females with binge‐eating disorder had more OC symptoms than normal weight females without binge‐eating disorder OC symptoms did not differ between overweight females and normal weight females with binge‐eating disorder OC symptoms could predict binge‐eating disorder in normal weight females, but not overweight females
Strahler et al. (2018)	General population *n* = 713 Females *n* = 569 (79.8%) Age *M =* 29.4, SD *=* 11.2 Germany	To examine whether orthorexia nervosa is of clinical relevance and can be distinguished from other mental health conditions	Yale‐Brown Obsessive Compulsive Scale Düsseldorf Orthorexia Scale	Greater OC symptoms observed in the orthorexia nervosa group compared to the non‐orthorexia nervosa group Compulsive behaviours, rather than obsessive symptoms, could predict orthorexia nervosa symptoms
Bóna et al. (2019)	Gym attendees *n* = 207 Females *n* = 140 (67.6%) Age *M =* 31.9, SD *=* 8.7 Hungary	To assess psychological correlates of orthorexia nervosa in gym goers	Maudsley Obsessive Compulsive Inventory Eating Disorders Inventory ORTO‐11 (Hungarian translation)	OC symptoms were not associated with orthorexia nervosa; eating disorder symptoms were associated with orthorexia nervosa
Łucka et al. (2019)	Students *n* = 864 Females *n* = 599 Age *M =* 20.21, SD *=* 3.27 Males *n* = 265 Age *M =* 18.93, SD *=* 3.67 Poland	To determine whether orthorexia nervosa is a part of, or separate to, OCD and eating disorders	ORTO‐15 Maudsley Obsessive Compulsive Inventory Eating Attitudes Test—26	No difference observed in OC symptoms between those at risk or not at risk of orthorexia nervosa OC symptoms of checking were associated with orthorexia nervosa symptoms
Zickgraf et al. (2019)	General population *n* = 449 Females *n* ≈ 220 (49%) *M =* 33.6, SD *=* 9.5 USA	To examine similarities and differences between orthorexia nervosa and eating disorder symptoms, such as OC symptoms	Obsessive‐Compulsive Inventory—Revised Nine‐Item ARFID Screen Eating Habits Questionnaire Clinical Impairment Assessment Eating Attitudes Test—26 (Severe restricting for thinness and binging and purging items)	OC symptoms were associated with eating disorder symptoms and orthorexia nervosa OC symptoms were only associated with the problems subscale of orthorexia nervosa when controlling for eating disorder symptoms, suggesting impairments associated with orthorexia nervosa may be similar to OCD, but not the eating behaviours themselves
Bartel et al. (2020)	General population and students *N* = 512 Females *n =* 423 (82.6%) Age *M =* 24.5 Canada	To explore the relationship between orthorexia nervosa, eating disorder symptoms and OC symptoms	Obsessive‐Compulsive Inventory—Revised The Revised Bratman's Orthorexia Test ORTO‐15 Eating Disorder Examination Questionnaire The Food Choice Questionnaire	OC symptoms correlated with orthorexia nervosa symptoms, eating disorder symptoms and food choices When controlling for eating disorder symptoms, the correlation between OC symptoms and orthorexia nervosa was reduced OC symptoms could predict orthorexia nervosa, but to a lesser extent than eating disorder symptoms
Brytek‐Matera et al. (2020)	General population *n* = 230 Females *n* = 175 (76.1%) Age *M =* 26.52, SD *=* 7.65 Poland	To determine psychopathological profiles of Polish adults concerning eating disorders, orthorexia nervosa and OC symptoms	Düsseldorf Orthorexia Scale Obsessive‐Compulsive Inventory—Revised The Three‐Factor Eating Questionnaire Eating Disorder Inventory	Three groups identified: (1) low psychopathology (2) high OC features, moderate eating disorder features/perfectionism (3) high eating disorder features/perfectionism, moderate OC features Participants with greater OC features were more likely to be at risk of orthorexia nervosa compared to those with low psychopathologies. However, those with moderate OC symptoms and greater eating disorder symptoms were more likely to have orthorexia nervosa
Dąbal and Dąbal (2020)	University students *n* = 236, of which: High orthorexia nervosa risk group *n* = 64 Females *n* = 34 (53.1%) Age *M =* 23.91, SD *=* 4.7 Low orthorexia nervosa risk group *n* = 172 Females *n* = 83 (48.3%) Age *M =* 24.97, SD *=* 4.56 Poland	Identify the relationship between orthorexia nervosa symptoms and psychopathological factors	ORTO‐15 Obsessive‐Compulsive Inventory—Revised	The relationship between OC symptoms and orthorexia nervosa approached significance OC symptoms were greater in the high orthorexia nervosa symptoms group compared to the low orthorexia nervosa symptoms group
Walker‐Swanton et al. (2020)	Participants reporting healthy eating/weight loss as primary dietary goal *n* = 130 Females *n =* 121 (93.1%) *M =* 21.03, SD *=* 7.67 Australia	Examine perceived need for treatment for orthorexia nervosa	Obsessive‐Compulsive Inventory—Revised Eating Disorder Examination Questionnaire—Short Version Eating Habits Questionnaire	OC symptoms significantly, positively correlated with eating disorder symptoms OC symptoms were associated with orthorexia nervosa, but not when controlling for eating disorder symptoms Those wanting treatment for orthorexia nervosa had greater OC symptoms than those not wanting treatment, but OC symptoms could not distinguish between those wanting or not wanting treatment
Zhou et al. (2020)	University students (total sample) *n* = 418 Females *n* = 219 (52.4%) Age *M =* 20.0, SD *=* 1.1 Males *n* = 199 (47.6%) Age *M =* 20.0, SD *=* 1.2 China	To examine correlates of orthorexia nervosa among Chinese university students	Obsessive‐Compulsive Inventory—Revised Orthorexia Nervosa Scale	OC symptoms were positively associated with orthorexia nervosa symptoms Those with elevated orthorexia nervosa symptoms had greater OC symptoms compared to those with non‐elevated orthorexia nervosa symptoms
Albertella et al. (2021)	General population *n =* 878 Females *n* = 466 (53.1%) Age *M =* 32.0, SD *=* 12.5 Australia, England, USA, Brazil	To examine the relationship between impulsive and compulsive traits and addictive behaviours during the COVID‐19 lockdown in Australia	Obsessive‐Compulsive Inventory—Revised Modified Yale Food Addiction Scale	An association between OC symptoms and food addiction was observed during lockdown
Barnhart et al. (2021)	Students *n =* 509 Females *n* = 390 (76.6%) Age *M =* 19.96, SD *=* 2.93 USA	To explore psychological wellbeing and picky/selective eating in students	The Short Obsessive‐Compulsive Screener Eating Disorder Examination Questionnaire Binge Eating Scale Adult Picky Eating Questionnaire Inflexible Eating Questionnaire	OC symptoms correlated with selective eating, eating disorder symptoms, binge‐eating, inflexible eating and social eating anxiety. The strongest correlation was between OC symptoms and selective/picky eating Inflexible eating and OC symptoms also predicted these eating behaviours
Novara et al. (2021)	University students with high orthorexia nervosa symptoms *n* = 43 Females *n* = 21 (48.8%) Age *M =* 21.44, SD *=* 2.02 University students with low orthorexia nervosa symptoms *n* = 259 Females *n* ≈ 152 (58.5%) Age *M =* 20.83, SD *=* 3.33 Italy	To consider whether orthorexia nervosa can be considered a distinct condition	Obsessive‐Compulsive Inventory—Revised Eating Disorder Inventory—Third Edition Eating Habits Questionnaire	Greater OC symptoms observed in the high orthorexia symptom group, compared to the low symptom group Eating disorder symptoms were linked to orthorexia nervosa when OC symptoms were controlled
Vanzhula et al. (2021)	Mixed student and eating disorder sample Students *n* = 1222 Females *n =* 617 (50.5%) *M =* 19.98 Eating disorders (sample 1) *n =* 168 Females *n =* 159 (94.6%) *M =* 26.27 Eating disorders (sample 2) *n =* 229 Females *n =* 217 (94.8%) *M =* 29.24 USA	To explore the relationship between OC symptoms, eating disorder symptoms and perfectionism	Obsessive‐Compulsive Inventory—Revised Eating Disorders Examination Questionnaire‐IV The Frost Multidimensional Perfectionism Scale	Perfectionism and obsessions link symptoms of OCD and eating disorders
Yakın et al. (2021a)	Students *n* = 921 Females *n* = 780 (84.6%) Age *M =* 20.72, SD *=* 2.63 France	To compare differences between those with OC symptoms, eating disorder symptoms and orthorexia nervosa symptoms	Obsessive‐Compulsive Inventory—Revised Eating Habits Questionnaire Eating Disorder Inventory—Third Edition (Drive for Thinness and Bulimia subscales)	Participants grouped into: OC symptom group, orthorexia nervosa symptom group, eating disorder symptom group, low psychopathology group Compared to the low psychopathology group, the OC group had greater orthorexia nervosa symptoms, although these were lower than the eating disorder group and orthorexia nervosa group The orthorexia nervosa group had similarly low levels of OC symptoms to the low psychopathology group, compared to the OC group The OC group had greater eating disorder symptoms than the low psychopathology and orthorexia nervosa groups, but this was lower than the eating disorder group
Yakın et al. (2021b)	Female students *n* = 362 Age *M =* 23.43, SD *=* 4.33 France	To explore profiles of female French students in regard to definitions of healthy eating, and examine these profiles with psychopathological symptoms	Obsessive‐Compulsive Inventory—Revised Eating Habits Questionnaire Eating Disorder Inventory—Third Edition (Drive for Thinness and Bulimia subscales)	OC symptoms not associated with orthorexia nervosa symptoms. OC symptoms were associated with eating disorder symptoms
Brytek‐Matera et al. (2022)	Polish students *n* = 286 Females *n* = 236 (82.5%) Age *M =* 22.33, SD *=* 2.38 Italian students *n* = 320 Females *n* = 255 (79.7%) Age *M =* 21.98, SD *=* 2.09 Italy and Poland	To examine the relationship between orthorexia symptoms and OC symptoms in students from Poland and Italy	Obsessive‐Compulsive Inventory—Revised Eating Habits Questionnaire	OC symptoms were weakly, but positively, correlated with orthorexia symptoms in Polish and Italian samples Those with greater orthorexia nervosa symptoms expressed more OC symptoms
Hallit et al. (2022)	General population *n* = 487 Females *n =* 372 (76.4%) Age *M =* 28.38, SD *=* 13.26 Lebanon	To examine orthorexia nervosa and OC symptoms in the Lebanese population	The 12‐item Obsession‐Compulsion Inventory Düsseldorf Orthorexia Scale Eating Attitudes Test—26	Greater OC symptoms were associated with orthorexia nervosa symptoms OC symptoms of washing were a unique predictor of orthorexia nervosa when controlling for eating disorder symptoms
Kinkel‐Ram et al. (2022)	General population *n =* 358 Females *n* = 141 (39.4%) Age *M =* 35.3, SD *=* 9.93 USA	To explore whether there is a longitudinal and bi‐directional relationship between OC symptoms and eating disorder symptoms	Yale‐Brown Obsessive Compulsive Scale Eating Disorder Examination Questionnaire version 6.0 Eating Disorder Examination Questionnaire—Short Version	Eating disorder symptoms and OC symptoms have a bi‐directional relationship over the course of 5 weeks. This was prominent for eating disorder symptoms and obsessions, rather than compulsions
Novara et al. (2022)	Dieters with high orthorexia symptoms *n* = 52 Females *n* = 28 (53.85%) Age *M =* 42.68, SD *=* 13.41 Dieters with low orthorexia symptoms *n* = 41 Females *n* = 24 (58.4%) Age *M =* 47.87, SD *=* 12.18 Non‐dieters with high orthorexia nervosa symptoms *n* = 40 Females *n* = 20 (50%) Age *M =* 20.78, SD *=* 2.71 Italy	To examine the role of dieting in those with high and low orthorexia tendencies, and their relationship with psychopathologies	Obsessive‐Compulsive Inventory—Revised Eating Habits Questionnaire—21	Levels of OC symptoms were similar across dieters, with or without orthorexia tendencies, and those with orthorexia nervosa that were not on a diet
Yang et al. (2022)	Female exercisers *n =* 295 Age *M =* 22.11, SD *=* 6.65 China	To examine personality traits and OC symptoms and their relationship with disordered eating among Chinese female exercisers	Obsessive‐Compulsive Inventory—Revised SCOFF The Ten‐Item Personality Inventory	OC symptoms were associated with eating disorder symptoms OC symptoms partially mediated the relationship between agreeableness, emotional stability and eating disorder symptoms, and fully mediated the relationship between conscientiousness and eating disorder symptoms
Yazkan and Uğurlu (2022)	Healthcare professionals *n* = 183 Females *n* = 129 (70.49%) Aged between 20 and 65 years Türkiye		Maudsley Obsessive Compulsive Inventory ORTO‐15	OC symptoms were positively associated with orthorexia nervosa symptoms
Barnhart et al. (2023) *Same participant sample as* Barnhart et al. (2021)	Students *n =* 509 Females *n* = 390 (76.6%) Age *M =* 34.70, SD *=* 10.50 USA	To explore the relationship between facets of picky/selective eating and psychological factors and quality of life	The Short Obsessive‐Compulsive Screener Eating Disorder Examination Questionnaire Binge Eating Scale Adult Picky Eating Questionnaire	OC symptoms were associated with poorer food variety, meal disengagement, meal presentation and taste aversion Pickiness over meal presentation and eating disorder symptoms could also predict OC symptoms
Dolapoglu et al. (2023)	Medical students *n* = 142 Females *n =* 75 (52.8%) Age *M =* 22 Türkiye	To understand the relationship between orthorexia nervosa, eating disorders and OC symptoms	Maudsley Obsessive‐Compulsive Inventory Eating Attitude Test—Short Form ORTO‐11	OC symptoms were not associated with orthorexia nervosa in medical students
Duradoni et al. (2023)	General population *n* = 587 Females *n* ≈ 506 (86%) Age *M =* 29.32, SD *=* 11.29 Italy	To examine orthorexia nervosa and OC symptom subtypes	Vancouver Obsessional Compulsive Inventory ORTO‐15 Orthorexia Nervosa Inventory	All OC symptom subtypes were associated with orthorexia nervosa, with OC obsessions being the strongest correlate
Levin et al. (2023)	University students *n* = 333 Females *n* ≈ 249 (72%) Age *M =* 20.9, SD *=* 4.3 Canada	To explore the relationships between orthorexia nervosa and associated psychopathologies	Obsessive‐Compulsive Inventory—Revised The Eating Habits Questionnaire Eating Disorder Examination Questionnaire	OC symptoms were correlated with orthorexia nervosa symptoms, but orthorexia nervosa symptoms shared a stronger relationship with eating disorder symptoms Those with orthorexia nervosa symptoms and eating disorder symptoms had elevated levels of OC symptoms, compared to those with less eating pathologies. Those with eating disorder symptoms had the highest OC symptoms
Fekih‐Romdhane et al. (2024)	General population *n* = 977 Females *n =* 753 (77.1%) *M =* 21.94, SD *=* 3.14 Poland, Lebanon, Italy	Examine the relationship between eating disorder symptoms, perfectionism and OC symptoms	Obsessive‐Compulsive Inventory—Revised The Obsessive Beliefs Questionnaire—44 Eating Attitudes Test—26 The Multidimensional Perfectionism Scale	Greater OC symptoms were associated with more eating disorder symptoms OC symptoms mediated the relationship between perfectionism and eating disorder symptoms, but perfectionism still had a direct effect on eating disorder symptoms
Greville‐Harris et al. (2024)	Students *n* = 507 Females *n* = 424 (83.6%) Age *M =* 22.23, SD *=* 8.7 England, UK	To examine the influence of perfectionism in the relationship between OC symptoms and perfectionism	Obsessive‐Compulsive Inventory—Revised Düsseldorf Orthorexia Scale (English version) Frost Multidimensional Perfectionism Scale‐Brief	Perfectionism partially mediated the relationship between OC symptoms and orthorexia nervosa symptoms OC symptoms had a direct effect on orthorexia nervosa when controlling for perfectionism
Huynh et al. (2024)	Undergraduate students *n* = 190 Females *n* ≈ 152 (80%) Age *M =* 28.63, SD *=* 9.88 Australia	To examine whether perfectionism is implicated in the relationship between OC symptoms and orthorexia nervosa	Obsessive‐Compulsive Inventory—Revised Obsessive Beliefs Questionnaire Eating Habits Questionnaire Clinical Perfectionism Questionnaire	OC symptoms could predict orthorexia nervosa symptoms Perfectionism moderated the relationship between OC symptoms and orthorexia nervosa symptoms
Latif and Moulding (2024)	General population *n =* 238 Females *n* = 161 (67.6%) Non‐binary/gender diverse *n* = 24 (10.1%) Age *M =* 29.32, SD *=* 10.47 Australia	To explore the effect of emotion regulation and feared self in the relationship between OC symptoms and eating disorder symptoms	Obsessive‐Compulsive Inventory—Revised Eating Disorder Examination Questionnaire Fear of Self—Multidimensional Version Difficulties in Emotion Regulation Scale Short Form	OC symptoms were not a significant predictor of eating disorder symptoms when controlling for other factors, including emotion regulation, depression, anxiety and gender
Rossi et al. (2024)	General population *n* = 1328 Females *n* = 976 (73.5%) Age *M =* 28.70, SD *=* 5.843 Italy	To examine whether worries about food and food preoccupation mediated the relationship between OC symptoms, eating disorder symptoms and orthorexia nervosa	Obsessive‐compulsive subscale of the Symptom Checklist‐90 Revised Eating Attitudes Test—26 ORTO‐15	OC symptoms correlated with eating disorder symptoms and orthorexia nervosa symptoms Worries about food/food preoccupation mediated the relationship between OC symptoms and orthorexia nervosa symptoms OC symptoms had a direct effect on orthorexia nervosa when controlling for worries about food
Zohar et al. (2025)	Students *n* = 772 Females *n* = 636 (82.4%) Age *M =* 29.40, SD *=* 8.50 Italy	To assess the relationship between adult picky eating, childhood picky eating and other variables including OC symptoms	Obsessive‐Compulsive Inventory—Revised Adult Picky Eating Questionnaire	Adult picky eating was positively associated with OC symptoms Childhood picky eating was associated with OC symptoms in adulthood

*Note:* ≈ Gender *n* based on percentages provided by the authors.

### 2.5 Synthesis of Results

In accordance with the JBI Manual for Evidence Synthesis, data from the eligible articles were extracted by the first author into a table to highlight core details of the studies (Aromataris et al. 2024). Any uncertainties in data extraction were discussed with the second author to ensure consistency and accuracy. The eligible articles were presented in a summary table detailing the study population, study aim, measures of pathological eating and OCD and/or OC symptoms, and key findings (Tables 1 and 2). A narrative summary of the findings was also provided. For this summary, the extracted data were reviewed and categorised into two themes based on the study population (i.e., the OCD population or general population). Within these themes, subthemes were organised according to the pathological eating behaviours observed:

Theme 1: Pathological eating behaviours within obsessive‐compulsive disorder.

- Eating disorders
- Non‐clinical eating behaviours

Theme 2: Obsessive‐compulsive symptoms and pathological eating behaviours in the general population.

- Eating disorder symptoms and obsessive‐compulsive symptoms
- Non‐clinical eating behaviours and obsessive‐compulsive symptoms

## 3 Results

### 3.1 Studies of the OCD Population

The participants in the study corresponded to 22,358 adults with OCD. Among OCD participants, there were a total of 12,421 females (note: some studies reported gender of the whole study sample, which may have included controls; this figure only includes studies which reported the number of females in OCD samples). Mean ages for participants in these studies ranged between 30.6 and 41.4 years and the studies were conducted across Europe, Asia, North America, South America and Oceania. Full details of each study can be found in Table 1.

All studies adopted quantitative methodology. In most studies, eating behaviours referred to the prevalence of eating disorders among those with OCD. Eating disorders and/or eating behaviours were generally assessed using diagnostic guidelines (e.g., Structured Clinical Interview for DSM‐IV axis I disorders; First 1997), variations of the Eating Disorder Questionnaire (Fairburn et al. 1993) or variations of the Eating Disorder Attitudes Test (Garner et al. 1982; Garner and Garfinkel 1979). Details of all instruments used are in Table 1.

### 3.2 General Population Studies

Studies of the general population which examined OC symptoms and eating behaviours consisted of 19,990 participants, of which 13,649 were female. The mean age of the participants ranged from 18.8 to 42.7 years and the majority of studies were carried out in Europe, North America and Asia. Details of each study are presented in Table 2.

All but one study used quantitative methodologies. The most used measures of OC symptoms were the Obsessive‐Compulsive Inventory—Revised (Foa et al. 2002) and the Maudsley Obsessive Compulsive Inventory (Hodgson and Rachman 1977). A wide range of measures to assess eating behaviours were used; the full list is provided in Table 2.

## 4 Scoping Review

### 4.1 Eating Behaviours Within Obsessive‐Compulsive Disorder

#### 4.1.1 Eating Disorders in Obsessive‐Compulsive Disorder

A high proportion of adults with OCD were reported to reach the threshold for an eating disorder, including anorexia nervosa, bulimia nervosa and binge‐eating disorder. For example, Danner et al. (2022) observed that 10.5% of OCD participants experienced an eating disorder at some point during their lifetime, with anorexia nervosa and binge‐eating disorder being the most frequently observed. Other researchers concur with the high rates of eating disorders reported in adults with OCD but note binge‐eating disorder to be the most prevalent (Torresan et al. 2013). In another study, M. T. Williams et al. (2017) observed no other eating disorder other than binge‐eating disorder (4.1%) among a sample of African Americans with OCD.

Individuals with OCD are also more likely to meet the criteria for a probable eating disorder compared to controls (Bang et al. 2020), with prevalence rates similar to what has been observed in those with other anxiety‐spectrum disorders (Tyagi et al. 2015). For example, Bang et al. (2020) demonstrated 22.6% of those with OCD, compared to 11.2% of healthy controls, surpassed the clinical cut‐off point for a likely eating disorder on the Eating Disorder Examination Questionnaire (EDE‐Q; Fairburn and Beglin 2011). However, sample means of OCD participant groups generally fall below the threshold required to meet a diagnosis of an eating disorder (Bang et al. 2020; Poyraz et al. 2015; Yılmaz et al. 2020).

Some studies have suggested that OCD should be considered a risk factor for developing an eating disorder. For example, a longitudinal study demonstrated those with OCD have an increased risk of developing anorexia nervosa, with males with OCD identified as particularly vulnerable (Cederlöf et al. 2015). Furthermore, Hofer et al. (2018) found that adolescents with OCD were specifically at a greater risk of developing bulimia nervosa. Collectively, these findings suggest that those with OCD have an increased risk of developing an eating disorder, even if there is a lack of agreement over which eating disorder poses the greatest risk.

### 4.2 Non‐Clinical Eating Behaviours

#### 4.2.1 Eating Disorder Symptoms

Eating disorder symptoms considered less severe and/or less frequent may not always warrant a diagnosis. Therefore, some studies have examined eating disorder symptoms within this population, rather than only those reaching the threshold for an eating disorder diagnosis. For example, Peters et al. (2019) observed that binging and compensatory purging behaviours were reported by 12.8% of participants with OCD. Moreover, Kaczkurkin et al. (2021) observed that, in a group of individuals with anxiety disorders, including OCD, there was a positive association between greater levels of OC symptoms and eating disorder symptomatology.

In comparison to healthy controls, adults with OCD have been shown to report more binging, purging, and compensatory behaviours to manage weight loss, and to score higher on overall measures of eating disorder symptoms. For example, those with OCD showing greater eating disorder symptomatology had more severe OC symptoms (Ay and Aytas 2018).

However, one particular study suggested that those with OCD do not differ in eating disorder symptomatology compared to healthy controls who lead a more active lifestyle, with both groups found to have increased levels of eating disorder symptoms compared to healthy controls who did not exercise (Yılmaz et al. 2020). Moreover, other studies have also found those with OCD are not at greater risk of eating disorder symptoms compared to some other clinical groups. For example, one study found that those with OCD display similar levels of eating disorder symptoms to those with anxiety disorders, such as generalised anxiety disorder and panic disorder (Poyraz et al. 2015). Hence, adults with OCD may not experience greater eating disorder symptomatology compared to other psychiatric groups.

Some studies have found that, whilst those with OCD may display eating disorder symptoms, other factors may underlie the expression of these symptoms. For example, Peters et al. (2019) observed that the predictive effect of OC symptoms on binge‐purge behaviours was reduced when controlling for mood instability and impulsivity. Similarly, Kaczkurkin et al. (2021) observed that depressive symptoms reduced the effect of OC symptoms on eating disorder symptoms. Therefore, it is possible that other factors are implicated in the relationship between eating disorder symptoms and OC symptoms.

#### 4.2.2 Orthorexia Nervosa

There have been mixed findings regarding the relationship between OCD and orthorexia nervosa, which refers to the desire to eat only pure and healthy foods. For example, one study reported that 2%–3.7% of inpatients with OCD presented with orthorexia nervosa (Hessler‐Kaufmann et al. 2021), and research by Poyraz et al. (2015) and Cosh et al. (2023) also demonstrated that increased levels of orthorexia nervosa symptoms in OCD corresponded to severe levels of OC symptoms. However, other studies have observed no relationship between symptoms of orthorexia nervosa and OC symptoms (Yılmaz et al. 2020), with those with OCD also found to report similar levels compared to other clinical populations, such as those with anxiety and depressive disorders, or healthy controls (Poyraz et al. 2015; Vaccari et al. 2021; Yılmaz et al. 2020).

Although it remains unclear whether levels of orthorexia nervosa may or may not be elevated in OCD specifically, there are some factors which might increase the likelihood of these eating behaviours in this population. For example, Yılmaz et al. (2020) demonstrated that individuals with OCD, who express greater levels of eating disorder symptomatology, such as anorexia nervosa and bulimia nervosa, were more likely to express symptoms of orthorexia nervosa. Similarly, in a group of individuals who self‐identified as dieters and/or having mental health symptoms (of whom 67% had OCD), symptoms of orthorexia nervosa were found to be associated more often with symptoms of eating disorder rather than OC symptoms (Cosh et al. 2023).

#### 4.2.3 Food Avoidance and Food Approach

Only one study has examined food approach behaviours in OCD patients. Rai et al. (2022) observed no differences between OCD participants and healthy controls regarding prevalence of food addictions. However, those with OCD, as a group, experienced more severe food addiction symptoms, characterised primarily by the compulsive consumption of palatable and hyperpalatable food items, compared to healthy controls. It is important to note that the Eating Attitude Test (EAT‐26; Garner et al. 1982) was used to assess food addiction, which is an assessment of eating disorders. As such, the findings of this study may be more reflective of bulimia nervosa or binging behaviours rather than food addiction per se.

Binging behaviours, which can be described as a food approach behaviour, have also been observed among those with OCD (Peters et al. 2019). However, no studies have addressed food avoidance in OCD, defined as all movements made away from food.

## 5 Obsessive‐Compulsive Symptoms and Eating Behaviours in the General Population

### 5.1 Eating Disorder Symptoms

#### 5.1.1 Anorexia Nervosa and Bulimia Nervosa

Studies of adults in the general population have noted that higher levels of OC symptoms correspond to more severe eating disorder symptoms associated with anorexia nervosa and bulimia nervosa. For example, those with elevated levels of OC symptoms are more likely to exhibit greater levels of dietary restraint, binging or purging (Barnhart et al. 2021; Fekih‐Romdhane et al. 2024; Gezer and Yalvaç 2018; Zickgraf et al. 2019). A further study by Kinkel‐Ram et al. (2022) also observed over a 5‐week period that eating disorder symptoms had a reciprocal relationship with OC symptoms of obsessions, but not compulsions.

Some studies have also observed that those expressing greater anorexia and/or bulimia nervosa symptoms are more likely to experience greater OC symptoms compared to typical eaters (i.e., those who think about food when they need to—when they're hungry or need to plan a meal) and selective eaters (Zickgraf et al. 2016). In contrast, individuals with clinically significant OC symptoms have been shown to exhibit similar low levels of pathological dieting behaviours to those without OC symptoms, casting doubt as to whether those with OC symptoms are more prone to eating disorder symptoms (Belloch et al. 2016). However, the same study did observe that those demonstrating clinically significant OC symptoms were likely to have intrusive thoughts about eating, a common finding amongst those with an eating disorder, such as anorexia nervosa.

Further research also proposed that specific eating disorder symptoms were associated with certain OC symptoms. For example, Pollack and Forbush (2013) observed that checking and cleaning OC symptoms could predict dietary restraint, and checking behaviours could predict binge‐eating behaviours. Moreover, in a separate study, which used a mixed sample of undergraduate students and adults with eating disorders, it was observed that secret binging and eating were associated with OC hoarding symptoms, and obsessive symptoms were related specifically to concerns about being seen eating in public (Vanzhula et al. 2021). These findings posit that those who present with certain OC symptoms may then be more at risk from different eating disorder‐related symptoms.

Importantly, some studies have also suggested that OC symptoms alone are not associated with eating disorder symptoms related to anorexia nervosa and bulimia nervosa. For example, Latif and Moulding (2024) demonstrated that OC symptoms could not predict eating disorder symptoms when controlling for other factors, including emotion regulation, depression and anxiety. Similarly, Pollack and Forbush (2013) observed that perfectionism and neuroticism mediated the relationship between OC symptoms and some eating disorder symptoms. Two other studies have also identified that OC symptoms mediate the relationship between perfectionism and eating disorder symptomatology, providing further evidence that several factors may be implicated in this relationship (Fekih‐Romdhane et al. 2024; Yang et al. 2022).

#### 5.1.2 Binge‐Eating Disorder

Binge‐eating symptoms, referring to uncontrollably consuming a large amount of food within a given time compared to the average person, have also been linked to OC symptoms. For example, Kim et al. (2018) reported greater OC symptoms in women with a healthy weight, engaging in binge‐eating behaviours, compared to their counterparts without binging behaviours. In comparison, overweight women engaging in binge‐eating had similar levels of OC symptoms to overweight women who did not binge. OC symptoms could also predict binge‐eating in women with a healthy weight, but not overweight women. Moreover, students with greater OC symptoms have been found to engage in more binging behaviours (Schulte 2016). However, whilst an initial association between OC symptoms and binge‐eating was observed, OC symptoms could no longer predict binge‐eating when controlling for emotional eating, indicating that again, other factors particularly those pertaining to fluctuating mood and emotions, are also implicated in this relationship.

#### 5.1.3 Avoidant‐Restrictive Food Intake Disorder

Two studies have examined the relationship between OC symptoms and ARFID in the general population. For example, Zickgraf et al. (2019) observed that greater levels of OC symptoms corresponded to more ARFID symptoms. Another study found that those with ARFID symptoms expressed high levels of OC symptoms comparable to those engaging in anorexia and/or bulimia nervosa eating behaviours, indicating that different presentations of eating disorder symptomatology are linked to greater levels of OC symptoms (Zickgraf et al. 2016).

### 5.2 Non‐Clinical Eating Behaviours

#### 5.2.1 Food Avoidance Behaviours

Food avoidance, specifically selective eating, characterised by the rejection of a wide range of foods leading to reduced food consumption and a less varied diet, has been observed in adults of the general population who display OC symptoms (Dovey et al. 2008). This has been evidenced through correlational analyses, where individuals reporting higher levels of OC symptoms express more selective eating behaviours (Barnhart et al. 2021; Zohar et al. 2025) and in group comparisons where adult selective eaters display significantly more OC symptoms compared to non‐selective eaters (Kauer et al. 2015; Zickgraf et al. 2016). Those with OC symptoms are also more likely to have selective preferences around meal presentation and disengage with meals, indicating that selectiveness extends to visual aspects of meals (Barnhart et al. 2023).

A qualitative study also observed patterns of OC symptoms among adult selective eaters (Fox et al. 2018). Here, participants noted that they viewed foods as ‘safe’ (i.e., foods deemed less dangerous) or ‘unsafe’, which would then lead to symptoms of anxiety and, subsequently, avoidance. These novel findings suggest that selective eaters may experience hyperfocus around the safety of certain foods, which may contribute to compulsive food avoidance.

#### 5.2.2 Food Approach Behaviours

Food approach, which refers to the desire to eat, has also been associated with OC symptoms. For example, Barnhart et al. (2021) found higher levels of binge‐eating behaviours to be associated with greater levels of OC symptoms. In a separate study, greater levels of food addiction were associated with increased levels of OC symptoms during the COVID‐19 lockdown period (Albertella et al. 2021). As the association was observed during novel circumstances, further research is needed to examine whether the relationship between food addictions and OC symptoms holds during more stable periods.

### 5.3 Orthorexia Nervosa

Some studies have suggested that adults of the general population who demonstrate orthorexia nervosa symptoms, the desire to eat pure and healthy foods, are more likely to have elevated levels of OC symptoms. For example, increased levels of orthorexia nervosa have been observed alongside OC symptoms (Brytek‐Matera et al. 2022; Bundros et al. 2016; Hayes et al. 2017; Levin et al. 2023; Yazkan and Uğurlu 2022; Zhou et al. 2020). More specifically, Łucka et al. (2019) observed that OC checking symptoms were associated with increased orthorexia nervosa behaviours, whereas other OC symptoms, such as cleaning or doubts, were not associated with orthorexia nervosa. OC symptoms were also noted to predict greater levels of orthorexia nervosa symptoms (Huynh et al. 2024). Hallit et al. (2022) demonstrated a unique predictive effect of OC washing symptoms on orthorexia nervosa, when controlling for other factors such as eating disorder symptoms. Another study by Strahler et al. (2018) found that compulsive symptoms, rather than obsessions, could predict orthorexia nervosa symptoms. Hence, it appears there is a relationship between orthorexia nervosa and OC symptoms, however there are mixed findings regarding which aspects of OC symptoms are related to this type of eating behaviour.

Further evidence for a link between OC symptoms and orthorexia nervosa is provided by between‐group studies. For example, Brytek‐Matera et al. (2020) and Yakın et al. (2021a) found that groups of those with OC features were more likely to be at risk of orthorexia nervosa or display orthorexia nervosa symptoms compared to those with low levels of psychopathology. Echoing these findings are studies which have demonstrated elevated levels of OC symptoms in those with orthorexia nervosa and/or related symptoms compared to those with lower levels of orthorexia nervosa symptomatology (Asil and Sürücüoğlu 2015; Brytek‐Matera et al. 2022; Dąbal and Dąbal 2020; Hayes et al. 2017; Novara et al. 2021; Strahler et al. 2018; Zhou et al. 2020). Interestingly both Asil and Sürücüoğlu (2015) and Dąbal and Dąbal (2020) failed to find an association between OC symptoms and orthorexia nervosa, but found that increased OC symptoms were present among participant groups characterised by greater levels of orthorexia nervosa symptoms, compared to those with fewer symptoms. Lastly, a study by Walker‐Swanton et al. (2020) observed that those wanting treatment for orthorexia nervosa had greater OC symptoms than those not wanting treatment, which may reflect the severity of orthorexia nervosa symptoms.

In contrast, some studies have failed to find a relationship between OC symptoms and orthorexia nervosa (Asil and Sürücüoğlu 2015; Bóna et al. 2019; Dąbal and Dąbal 2020; Dolapoglu et al. 2023; Yakın et al. 2021b). Interestingly, an earlier study by Brytek‐Matera et al. (2017) observed no relationship between OC symptoms and orthorexia nervosa in males, and a negative relationship in females, where increased OC symptoms were associated with fewer orthorexia nervosa symptoms. Moreover, Łucka et al. (2019) observed that OC symptoms were similar between those at risk of orthorexia nervosa and those without such risk. OC symptoms in an orthorexia nervosa participant group were also similar to participants with low levels of psychopathology (Yakın et al. 2021a). Additionally, Novara et al. (2022) observed that OC symptoms did not differ between three participant groups, including those with orthorexia nervosa and those engaging in an active diet, with or without orthorexia nervosa tendencies. These results suggest that OC symptoms may not be elevated in those with orthorexia nervosa compared to other groups.

The mixed findings regarding the relationship between orthorexia nervosa and OC symptoms may be partly explained by alternative variables. For example, whilst OC symptoms seem to be associated with orthorexia nervosa, some studies have observed that this relationship is reduced, or even negated, when considering eating disorder symptoms relating to anorexia nervosa or bulimia nervosa, suggesting that orthorexia nervosa should be considered a subcategory of eating disorders, rather than OCD (Bartel et al. 2020; Novara et al. 2021; Rossi et al. 2024; Walker‐Swanton et al. 2020; Zickgraf et al. 2019). Moreover, perfectionism, a core characteristic of OCD as well as eating disorders, has also been implicated in the relationship between OC symptoms and orthorexia nervosa as a mediator or moderator (Greville‐Harris et al. 2024; Huynh et al. 2024). However, it is also important to note that some studies, which have controlled for alternative variables such as perfectionism and eating disorder symptoms, have continued to observe a relationship between OC symptoms and orthorexia nervosa, highlighting the complexity of this relationship (Greville‐Harris et al. 2024; Hallit et al. 2022; Rossi et al. 2024).

## 6 Discussion

Overall, the findings from the literature review demonstrate some association between OC symptoms and pathological eating behaviours in the clinical OCD population, with some adults with OCD appearing to be at greater risk of developing eating disorders, particularly dieting related eating disorders and/or symptoms (i.e., anorexia nervosa and bulimia nervosa; e.g., Bang et al. 2020; Garcia et al. 2020). Fewer studies were found to have investigated non‐clinical eating behaviours in OCD participants, however there was some evidence to suggest that food approach behaviours and orthorexia nervosa may present in some adults with OCD. Similar findings were also observed among the general population, whereby endorsement of pathological eating behaviours was found to be associated with elevated levels of OC symptoms, particularly in the presence of emotion and mood difficulties.

Research has primarily focused on eating disorders associated with dieting (i.e., anorexia nervosa and bulimia nervosa). However, there is evidence to suggest that two disorders at opposite end of the restrictive food intake spectrum are associated with OCD. For example, binge‐eating disorder, characterised by uncontrollable episodes of eating, and ARFID, characterised by extreme food avoidance, have been linked to OCD (Kim et al. 2018; M. T. Williams et al. 2017; Zickgraf et al. 2016). Research has highlighted that as many as 4%–20% of ARFID patients may experience comorbid OCD or OC symptoms, at rates which are not dissimilar to other eating disorders, such as anorexia nervosa (Bryson et al. 2018; Fisher et al. 2014; Zickgraf et al. 2016). Therefore, OCD may not be uniquely related to dieting‐related disorders, but a range of pathological eating disorder patterns, including ARFID. However, there are no studies which have explored the prevalence of ARFID in OCD, specifically.

Despite those with OCD experiencing more intense OC symptoms during daily routines, including when eating at mealtimes (Brierley et al. 2021), few studies to date have highlighted a relationship between OC symptoms and non‐clinical eating behaviours, such as food avoidance, which encompasses selective eating and food neophobia (Rai et al. 2022; Zickgraf et al. 2016). Given that these non‐clinical eating behaviours have been considered a risk factor for developing an eating disorder in later life, there is a need to better understand and identify non‐clinical eating behaviours earlier (Derks et al. 2024; Herle et al. 2020).

Most studies to date have taken a broad approach to exploring OC symptoms and pathological eating behaviours. However, OCD and its symptoms are complex and have varied presentations; for example, one individual with OCD may experience obsessions and compulsions regarding contamination, whereas another may experience symptoms involving symmetry, orderliness, or religion (Moreno‐Amador et al. 2023; Van Schalkwyk et al. 2016). Although limited, there is some evidence to suggest specific OC symptoms are associated with certain eating behaviours. For example, OC checking symptoms appeared to be associated with orthorexia nervosa, dietary restraint and binging, OC contamination symptoms shared a relationship with dietary restraint (Pollack and Forbush 2013; Poyraz et al. 2015) and OC hoarding and obsessing were associated with concerns about being seen eating in public (Vanzhula et al. 2021). Not all individuals with OCD or OC symptoms display pathological eating behaviours, hence it would be of interest to further explore whether subsets of those with OCD are more at risk from specific eating concerns.

It is possible that similar characteristics between those with OCD or an eating disorder may underlie the expression of eating disorder symptoms in OCD, however further research is needed to explore this relationship. For example, some research has posited that perfectionism or difficulties with emotion regulation may account for the association between OC symptoms and pathological eating behaviours in both the OCD population and general population (Kaczkurkin et al. 2021; Latif and Moulding 2024; Pollack and Forbush 2013; Yang et al. 2022). These findings are of interest as perfectionism and emotion regulation are recognised risk and maintenance factors for eating disorders, as well as characteristics of the OCD population (Calkins et al. 2013; Fairburn et al. 2003; Maia et al. 2009; B. M. Williams and Levinson 2021). Moreover, there would be merits in exploring whether there are factors beyond perfectionism and emotion regulation which underlie the relationship between pathological eating behaviours and OCD or OC symptoms, such as sensory sensitivity or cognitive rigidity as proposed by other studies in both neurotypical and neurodiverse populations (Farrow and Coulthard 2012; Zickgraf et al. 2022).

In summary, the literature suggests that OC symptoms across both general and clinical OCD populations are linked to pathological eating behaviours. Given that non‐clinical eating behaviours, such as food selectivity, and eating disorders have been shown to have significant psychological, social, and functional limitations (Fildes et al. 2015; Galloway et al. 2003; Galloway et al. 2005; Wildes et al. 2012), there is a need to acknowledge that pathological eating patterns often present among those with OCD and/or displaying high levels of OC symptoms. It would also be important to disentangle the relationship between OC symptoms and pathological eating as this could further inform interventions. Although several studies reported associations between OC symptomatology and atypical eating behaviours, it remains unclear whether these patterns are specific to OCD or reflect broader overlaps with eating disorders and transdiagnostic features, such as perfectionism and emotion regulation. As research which addresses the nature of eating pathologies in OCD is limited, future studies should examine whether such eating behaviours are secondary to OCD or OC symptoms, or stem from shared transdiagnostic processes.

## Author Contributions

**Sonay Kucukterzi‐Ali:** conceptualization, data collection, formal analysis, methodology, writing – original draft, review and editing. **Amanda K. Ludlow:** conceptualization, formal analysis, methodology, writing – review and editing. **Roberto Gutierrez:** conceptualization, writing – review and editing. **Naomi A. Fineberg:** conceptualization, writing – review and editing. **Tim M. Gale:** conceptualization, writing – review and editing.

## Funding

The authors have nothing to report.

## Ethics Statement

The authors have nothing to report.

## Conflicts of Interest

The authors declare no conflicts of interest.
